# Socially Acquired Nocebo Effects Generalize but Are Not Attenuated by Choice

**DOI:** 10.1093/abm/kaad056

**Published:** 2023-09-27

**Authors:** Cosette Saunders, Ben Colagiuri, Kirsten Barnes

**Affiliations:** School of Psychology, University of Sydney, Sydney, Australia; School of Psychology, University of Sydney, Sydney, Australia; School of Psychology, University of New South Wales, Sydney, Australia

**Keywords:** Nocebo, Social modeling, Choice, Generalization, Virtual Reality, Cybersickness

## Abstract

**Background:**

Socially observing a negative treatment-related experience has been shown to modulate our own experience with the same intervention, leading to worsened health outcomes. However, whether this social learning generalizes to similar but distinct interventions has not been explored nor what manipulations can reduce these effects.

**Purpose:**

To determine whether socially acquired nocebo effects can be generated by observing a negative experience with a similar, but distinct intervention, and whether choice can reduce these effects.

**Methods:**

Across three experiments, a community sample of healthy adults (*N* = 336) either watched a confederate report cybersickness to the same Virtual Reality (VR) activity they were assigned to (Social Modeling: Consistent); a similar, but different VR activity (Social Modeling: Inconsistent); or did not view the confederate (No Social Modeling). Participants were either given choice over the VR (Choice) or assigned by the experimenter (No Choice).

**Results:**

Across the experiments, there was significantly greater cybersickness in both Social Modeling groups relative to No Social Modeling, while the two Social Modeling groups did not differ. There was no significant effect of Choice or a Choice by Social Modeling interaction. Social Modeling elicited greater anxiety and expectancies for cybersickness. Furthermore, these mechanisms mediated the association between social modeling and cybersickness.

**Conclusions:**

Socially acquired side-effects were demonstrated to generalize to similar, but distinct interventions, highlighting the diffuse and robust effect social modeling can have on our experiences. However, choice did not attenuate the experience of cybersickness, highlighting the need for alternative methods to counteract the effect of social modeling.

## Teaser

Witnessing someone experience cybersickness during Virtual Reality (VR) generated a nocebo effect in the observer, exacerbating their own symptoms when subsequently encountering VR. The nocebo effect was not specific to the VR activity witnessed, but generalised across different VR experiences, demonstrating that socially acquired nocebo effects are likely to spread rapidly.

## Introduction

Our prior beliefs and experiences are known to modulate our perception of the world, including the experience of symptoms and side-effects [1]. A primary route through which these symptom-related expectancies are formed concerns the observation of others [2]. For example, witnessing another individual experience side-effects to a treatment can increase side-effect reporting in the observer when the same treatment is subsequently encountered [3–5]. These socially acquired nocebo effects have been documented across a diverse range of symptoms, including pain, headache, and nausea [3, 4, 6–11]. Furthermore, they have significant clinical and societal ramifications given that socially propagated symptoms of this type have been demonstrated to spread at the community level [12].

Somewhat surprisingly, however, studies have yet to investigate: (i) how social information regarding symptoms generalize across similar contexts and interventions, and; (ii) how nocebo effects generated through social modeling (i.e., observing another individual) can be attenuated or blocked. These factors are of importance. As discussed below, not only do they provide novel information about the way socially modeled symptoms are transmitted, but also potential routes to mitigate the burden of nocebo effects.

At present, we know that the social acquisition of negative health outcomes can occur with identical interventions and treatments (e.g., [3, 4, 13]). Of even greater concern, is that socially modeled symptoms may generalize (i.e., spread) to other experiences. That is, observing a person experience a negative outcome to a specific experience or treatment, may not only cause the observer to experience nocebo effects to that specific experience, but also to other similar experiences. Hypothesizing that socially acquired nocebo effects might generalize in this manner is not unfounded. While not concerned with socially modeled symptoms, nocebo effects induced via direct classical conditioning have been demonstrated to generalize across environmental contexts. For example, participants conditioned to expect nausea from active Galvanic Vestibular Stimulation (GVS), experienced similar levels of nocebo nausea at test, irrespective of whether sham-GVS was delivered in the same room or a different context [14]. Given the mechanisms underlying the nocebo effect have been suggested to be similar across modes of induction (i.e., conditioning, social modeling, and explicit instruction) [15], it is of interest to determine whether socially modeled symptoms can generalize beyond identical interventions, as well as confirm the underlying mediators of any such effect.

Furthermore, given the documented strength of socially modeled nocebo effects [2], it is important to find interventions that can reduce these maladaptive health outcomes. Choice over treatment [16], side-effect framing [17, 18], nocebo education [19–22], latent inhibition [23, 24], and affect manipulations [25] have been proposed as interventions to reduce nocebo effects. A recent meta-analysis established choice as an effective method to enhance placebo effects. As such, choice may be a promising avenue through which to attenuate the nocebo effect, although research in this area has yet to be applied to social modeling [26]. Choice is inherently desirable [27, 28], and thus presents a low cost, nondeceptive, and ethical intervention [16, 29], previously shown to facilitate the placebo effect with respect to pain, discomfort, and sleep [26, 29–31]. Leotti et al. [28] contend that choice is a vehicle for individuals to exercise control, and thereby facilitate a reduction in anxiety and improve mental and physical outcomes [32, 33]. Alternatively, choice may engender a positive affect which could increase placebo effects and reduce nocebo effects [25, 27]. To date, however, only one published study has investigated choice with respect to the nocebo effect [16]. Here, participants who were given choice between two supposed betablockers (actually placebos) reported lower anxiety and fewer side-effects than those assigned to one of the supposed betablockers without any choice. As such, choice provides a potential but untested route through which to diminish socially modeled symptoms.

In order to bridge these two gaps in the literature, the present study investigated the effects of generalization and choice on socially acquired symptoms across three experiments all employing a common methodology. To achieve this a novel Virtual Reality (VR) model was implemented to investigate cybersickness; a constellation of nausea-related symptoms [34] previously reported to be susceptible to social modeling [35]. To explore generalization, participants witnessed a confederate experience cybersickness resulting from a VR activity that was either the same (i.e., Social Modeling Consistent: Confederate undertakes a rollercoaster ride) or different (i.e., Social Modeling Inconsistent: Confederate undertakes aerobatics) to the one that they subsequently experienced. The perception of choice was manipulated by allowing half of the participants to select a VR environment (i.e., choice of sunny or snowy weather) and yoking the remaining participants to their choices. Regardless of the participant’s choice, the same VR activity (i.e., the rollercoaster ride) was undertaken by all participants. Finally, expectancy, anxiety, control, and affect were measured throughout the experimental session, allowing for an exploration of potential underlying mechanisms of social modeling and choice.

Past research has repeatedly shown an effect of social observation on the nocebo effect for identical interventions and treatments [3, 4, 6, 7, 35, 36]. Therefore, it was hypothesized that participants assigned to the social modeling groups would report higher levels of cybersickness subsequent to VR exposure when compared with those who received No Social Modeling. This effect of social modeling was expected to generalize, occurring even when participants believed they were viewing a model experience symptoms in a VR environment different to their own. We anticipated that participants given choice over their VR environment would report lower levels of cybersickness across all social modeling conditions [16]. Given a paucity of empirical evidence concerning the role of choice with respect to socially modeled nocebo effects, there was no basis for directional hypotheses and as such interactions between social modeling and choice were explored. Evidence for the influence of psychological factors that mediate socially acquired nocebo effects is inconclusive [1, 8, 10, 35, 37]. Consequently, state anxiety, expectancy, control, and affect were explored as mediators of the social modeling and choice effects. We present details regarding the primary outcome of each of the three experiments in the text (with secondary analyses in Supplementary Materials) and then a pooled analysis of the combined data across the three experiments.

Experiment 1 examined both generalization and choice. Counter to hypotheses, observers who witnessed a social model undergo a different VR experience to the one they subsequently encountered, reported greater cybersickness than those who witnessed a social model undergo the same experience as their own. To assess the validity of this unexpected result of generalization, Experiment 2 focused specifically on generalization independent of choice, finding the nocebo effect to be equivalent across social modeling conditions. Given the discrepant results regarding generalization in Experiments 1 and 2, Experiment 3 was run to adjudicate, while adding choice back into the model to explore its impact on the nocebo effect. Finally, a pooled analysis was run that combined data from all experiments.

## Experiment 1

### Methods

Experimental design and analyses were preregistered (AsPredicted #68104). Ethical approval was granted by the University of Sydney Human Research Ethics Committee (Protocol 2021/301). The recruitment process for all experiments is depicted in Supplementary Material 1. Data collection occurred between June 30 and August 20, 2021.

#### Participants

Participants (*N* = 134) were recruited Australia-wide via Facebook advertisements. The sample comprised of 68 males and 66 females, 18–58 years of age (*M* = 32.20, *SD* = 7.06). Information regarding sample race and socioeconomic status (SES) were not collected. In keeping with our previous research [17, 35, 38], participants were ineligible if they had experienced VR > 10 times, had a medical condition increasing postural instability or risk of nausea, were pregnant, had epilepsy, a pacemaker, or preexisting binocular visual abnormalities. Access to a smartphone with diagonal screen size of 4.7–6.4 inches and able to run the latest YouTube application was necessary for participation. All participants were provided with a VR headset which they kept as compensation for their time.

#### Design

The current study was presented to participants as an investigation regarding online learning in VR. However, the true purpose was to examine the role of choice and generalization with respect to socially acquired nocebo nausea using a VR-based cybersickness model. Testing took place via the video-conferencing application Zoom, with all participants experiencing a VR rollercoaster ride. A 3(Social Modeling: No Social Modeling, Consistent, Inconsistent) × 2(Choice: No Choice, Choice) between-subjects design was employed. Participants were randomly assigned (via random number generator) to experimental condition and type of VR environment using a gender-stratified yoking procedure based on their order of participation. Full details are provided in Supplementary Material 2. The primary outcome was the severity of symptoms previously modeled by the confederate (general discomfort, nausea, and stomach awareness). State measures of anticipatory anxiety and expectancy were measured as potential mediators of virtually transmitted cybersickness.

##### Social modeling manipulation

Those randomly assigned to the social modeling groups watched an actor (who they believed was a real participant) experience VR and report symptoms of cybersickness before they experienced VR themselves. In line with previous social modeling research [3, 4, 13] those in the “Consistent” condition observed the actor describe the same VR activity that they chose/were assigned to (i.e., a rollercoaster ride). Importantly, those in the “Inconsistent” condition watched a description of a different environment and activity from their own (i.e., VR Aerobatics). Evidence of similar increased symptom reporting in both the Inconsistent and Consistent groups, relative to control, is therefore indicative of the generalization of a socially modeled nocebo effect across VR activities. The control group did not witness any social modeling prior to their VR experience. The two VR environments were selected from pilot data (*N* = 28), where five VR activities were paired with four environmental conditions. Preference for the snowy versus sunny environment, and rollercoasters vs. aerobatics did not differ (both *p*s > .05).

##### Choice

Participants assigned to the Choice condition chose which of two VR videos they preferred to watch. To control for differences in cybersickness that could be induced by substantial differences in the content of the VR video (e.g., a rollercoaster vs. aerobatics), participants chose their VR video based on peripheral environmental features (i.e., the weather; a snowy or sunny day). Unbeknownst to participants, the primary property of the VR that may provoke cybersickness (i.e., the rollercoaster ride) was constrained, while they were led to believe that they had chosen this (i.e., “you chose the snowy environment, which is a rollercoaster”). This isolated the manipulation to the perception of choice alone and controlled for the endogenous features of the VR. A manipulation of this type is similar to existing choice studies regarding the placebo effect that typically administer identical sham-treatments with peripheral perceptual differences [26, 29]. Manipulation of these peripheral environmental features (e.g., brightness/contrast associated with the different environments) should not modulate the induction of cybersickness [39].

#### Materials and measures

##### Social model

Social modeling took place virtually with live video interactions occurring via webcam on Zoom. While the participant believed the social model to be another participant (referred to as “Julian”) who was present in the Zoom session, interactions with the model were actually a series of pre-recorded videos of a male confederate. Open Broadcasting Software was used to pipe the pre-recorded film of the social model into Zoom. This ensured that all participants within each experimental condition were presented with identical social information. The scripts delivered by the confederate were indistinguishable between conditions except for a single reminder concerning the environment (i.e., sunny or snowy), and two reminders of the activity (i.e., rollercoaster or aerobatics). The videos contained subtle cues (e.g., the model looking to the left) that allowed the experimenter to “talk” to the confederate, creating the illusion that he was participating in real time. Only one participant (0.7% of the sample) reported being aware that the model was not real in a post-experiment manipulation check. During the actor’s modeling phase, they verbalized and gestured three distinct symptoms of cybersickness (nausea, general discomfort, and stomach awareness). The modeled symptoms were selected from pilot data in the lab so that they were those most likely to occur from VR exposure. See https://youtu.be/AcTfjDa4AjY for an excerpt of an experimental session, containing the script the confederate delivered as he modeled symptoms.

##### VR headsets and environments

Participants experienced VR using a Shinecon VR G034A head-mounted display. All participants watched one of two VR videos that depicted the same rollercoaster on either a sunny day (see https://youtu.be/19tjefId4oE) or a snowy day (see https://youtu.be/M9Vc-AT-TeM) which served as the “cybersickness-inducing” stimulus. In reality, the short rollercoaster ride served as a plausible activity to experience cybersickness while minimizing the occurrence of cybersickness due to the VR video alone. The videos were created using the custom rollercoaster simulator software NoLimits2 [40] which allowed for the generation of identical videos in length and content while only manipulating the environmental features that participants experienced. Screenshots are presented in Supplementary Material 3.

#### Primary outcome

##### Cybersickness

Cybersickness was measured using the 16-item Simulator Sickness Questionnaire (SSQ) [41]. Symptoms were rated on an 11-point scale: 0 (*not at all*) to 10 (*severely*). A baseline measure was taken at the beginning of each session, with the active measure taken immediately after VR. The difference score (active minus baseline) was used to measure cybersickness. Given three symptoms of the SSQ were specifically modeled to participants (general discomfort, nausea, and stomach awareness), cybersickness severity based on these items (referred to hereafter as “Modeled-SSQ”) formed the primary outcome, with the full sum-scored SSQ preregistered as a secondary outcome (“Full-SSQ”).

#### Secondary outcomes

Analysis of secondary outcomes are presented as Supplementary Materials.

##### Expectancy

Expectancy was assessed with the single item: “How much do you expect to experience feelings of cybersickness (e.g., nausea, general discomfort, stomach awareness) during the VR video?,” with an 11-point scale: 0 (*not at all*) to 10 (*severely*).

##### State anxiety

The short-form State-Trait Anxiety Inventory (STAI-6) [42] was employed with a 4-point rating scale: 1 (*not at all*) to 4 (*very much*). The overall STAI-6 sum score was calculated as the outcome.

##### VR anxiety

VR-specific state anxiety was measured via the single item: “How anxious do you feel about experiencing the Virtual Reality video?,” with the same scale as expectancy.

Baseline measures of secondary outcomes were recorded at the beginning of the study session, and active measures immediately prior to the rollercoaster experience (i.e., following the observation of the social model in the Social Modeling groups). Higher scores indicated greater baseline-adjusted expectancy, state anxiety, and VR anxiety.

##### Control and affective state

Control and affect were measured via the Self-Assessment Manikin (SAM) [43], with a 9-point pictorial scale [44]. The SAM included the dimensions of valence (unhappy/happy), arousal (calm/agitated), and dominance (controlled/in control). Measurement occurred immediately after participants chose/were assigned their environment. Higher scores indicated a more positive valence, greater arousal, and more perceived control.

##### Manipulation check

A manipulation check was implemented at the end of each experimental session. Participants were asked to provide an open response to the question “Briefly describe (1–2 sentences) what you thought the purpose of this experiment was”:

#### Procedure

Refer to Supplementary Material 4 for a diagram of experimental procedure. Participants were first screened for eligibility via Qualtrics. Ineligible participants were directed out of the signup process and eligible participants proceeded to the Participant Information Statement and Consent form. Upon consent, participants completed a short demographic survey. Participants were mailed the Headset 1–2 days prior to their scheduled experimental session.

Participants attended the experiment via Zoom, with the same female experimenter and male social model (actually pre-recorded footage). Participants received study information and completed baseline measures via Qualtrics. To maintain the cover story and avoid suspicion concerning repetition of questions regarding their wellbeing, participants were informed that monitoring their wellbeing throughout the experiment was an ethical requirement of the study. Then, all participants viewed information regarding the two VR environments (location—i.e., sunny environment presented on the right or left of the screen—was counterbalanced across participants). Those in the Choice conditions chose their preferred environment, while those in the No Choice conditions were told to click on their assigned environment (controlling for differences in the experience of agency).

All participants then completed the SAM to measure their sense of control and affect after VR selection. Participants in the No Social Modeling condition also completed a 5-min distractor task consisting of spatial reasoning questions (Raven’s progressive matrices [45]) aligning the timing of the VR experience between Social Modeling and No Social Modeling groups. This ensured that there was no confounding effect of time on anxiety and expectancy ratings, which may be stronger toward the beginning of the experimental session [35]. Those in the social modeling groups were told the confederate would experience VR first. Depending on the participant’s chosen or assigned environment, those in the Social Modeling Consistent condition were told: “both you and Julian [the confederate] chose/were assigned to experience the ‘sunny day’ environment, which contains a rollercoaster ride,” while those in the Social Modeling Inconsistent group were told “You chose/were assigned to experience the ‘sunny day’ environment which contains a rollercoaster ride, while Julian, you chose/were assigned to experience the ‘snowy day’ environment which contains ‘aerobatics’.” These two groups subsequently witnessed the confederate describe the VR environment including the experience of general discomfort, nausea, and stomach awareness (i.e., modeled symptoms). The experimenter then asked the confederate: (i) Could you describe your experience of that VR video?; and (ii) Can you tell me how you feel (physically) at the moment? The confederate responded with the modeled symptoms. After observing the confederate, participants in the social modeling groups completed the active expectancy and anxiety measures. They then underwent the VR experience themselves, with the confederate “watching.” Those in the “No Social Modeling” group undertook the same procedure but in reverse (i.e., they underwent the VR experience first while the confederate watched them and then watched the confederate undergo the VR experience). Participants answered the same two questions regarding their VR experience as the confederate (i.e., description and symptoms) and completed the active SSQ measure, several bogus memory questions to uphold the cover story, and the manipulation check. Upon completion participants were thanked for their time and informed they would receive a debriefing statement via email once data collection was complete.

#### Power and data analysis

Sample size was determined a priori. A minimum of 22 participants per-group were required to achieve 80% power (α = 0.05) with an effect size of η_*p*_^2^ = 0.07 (based on the effect size of choice reported by Bartley et al. [16], the hypothesized smaller effect of the two manipulations).

Participants with extreme baseline cybersickness (prespecified as 3 *SD* above the mean, *N* = 3) were excluded from analysis. Participants that failed the manipulation check (by explicitly noting that the social model was not a real participant) were also excluded from the analysis (*N* = 1). Participants were also excluded due to technical difficulties, not following instructions, or withdrawal (*N* = 7).

The primary analysis was a two-way ANCOVA with the between-subjects factors of social modeling (Social Modeling Consistent, Social Modeling Inconsistent, and No Social Modeling) and choice (Choice and No Choice) as the independent variables and baseline-adjusted Modeled-SSQ score as the dependent variable (active measure minus baseline). Gender was entered as a covariate, as it has previously been shown to moderate socially acquired nocebo effects [7]. Two planned orthogonal contrasts were conducted: (i) social modeling groups (Social Modeling Consistent and Inconsistent, combined) versus control (No Social Modeling); (ii) Social Modeling Consistent versus Social Modeling Inconsistent. Those in the groups that were given choice were compared with those without choice. Unless otherwise specified, all statistical analyses were performed using R version 4.0.3 [46]. The significance value for all tests was set at an alpha rate of .05.

### Results

There were no significant between-group differences in age, baseline measures (SSQ, state anxiety, VR anxiety, and expectancy), gender, or prior VR experience (all *p*s > .05), see Supplementary Material 5.

**Fig. 1.**
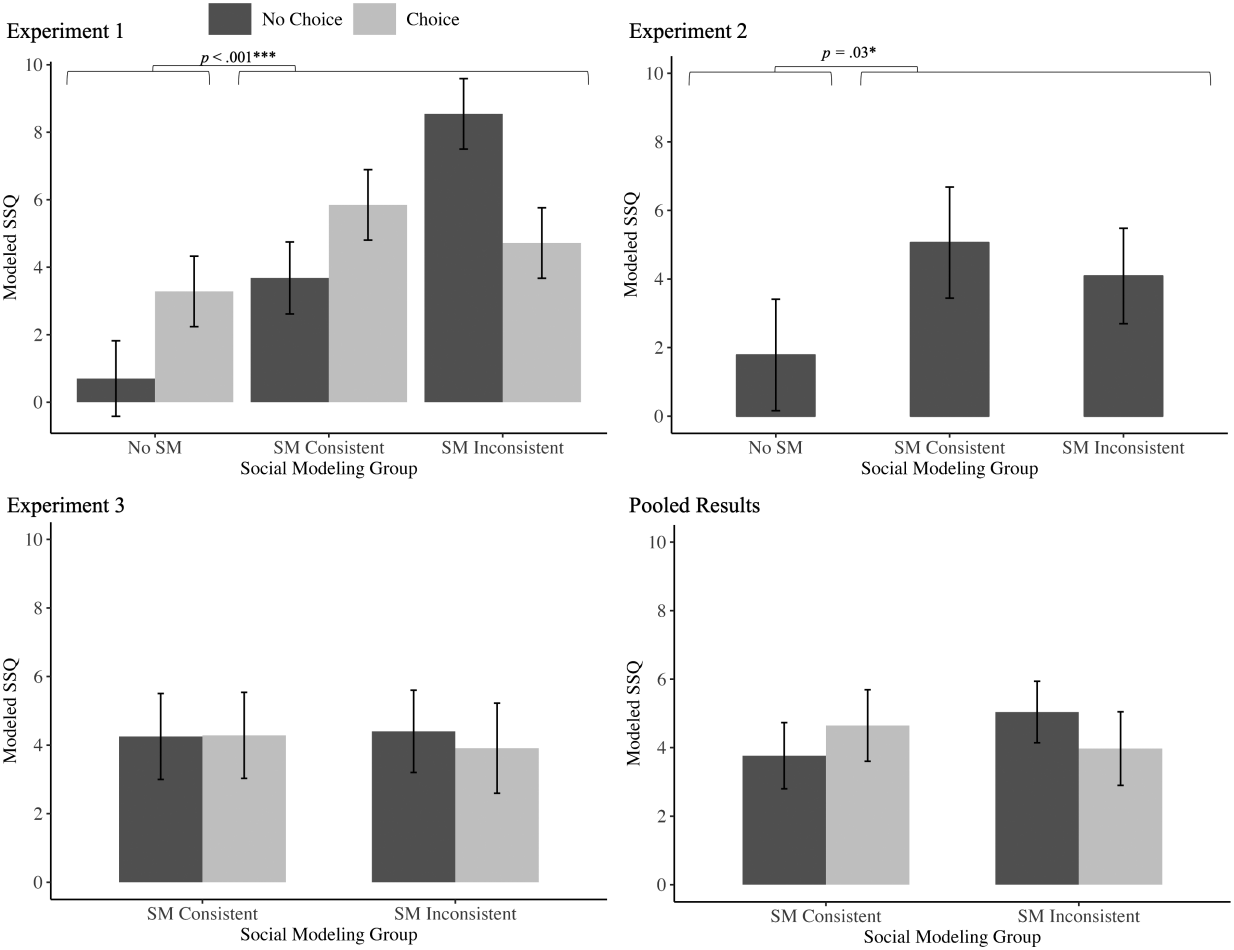
Mean baseline-adjusted Modeled-SSQ score by group for Experiments 1, 2, and 3. *Note.* All error bars are ± 1 SEM and account for the covariate (gender). Significant main effects are highlighted. Refer to text for significant interaction effects.

Figure 1 depicts the group means for the primary outcome. The ANCOVA model revealed that there was no main effect of the covariate, gender *F*(1,127) = 1.44, *p* = .23, η_*p*_^2^ = 0.01, nor was there a main effect of choice *F*(1,127) = 0.12, *p* = .72, η_*p*_^2^ < 0.01. However, a significant main effect of social modeling was observed, *F*(2,127) = 9.60, *p* < .001, η_*p*_^2^ = 0.13 which was qualified by a social modeling × choice interaction *F*(2,127) = 5.79, *p* = .004, η_*p*_^2^ = 0.08. Planned orthogonal contrasts revealed that Modeled-SSQ scores were elevated in the Social Modeling, relative to No Social Modeling, conditions *F*(1,127) = 15.94, *p* < .001, η_*p*_^2^ = 0.11. However, there was no significant overall difference in Modeled-SSQ scores between the Consistent and Inconsistent groups when collapsed across Choice, *F*(1,127) = 3.16, *p* = .08, η_*p*_^2^ = 0.02. There was no significant interaction between Choice and the orthogonal contrast that compared Social Modeling to No Social Modeling conditions on Modeled-SSQ scores, *F*(1,127) = 3.38, *p* = .07, η_*p*_^2^ = 0.03. However, there was an interaction between Choice and the second orthogonal contrast assessing generalization (i.e., comparing Consistent vs. Inconsistent Modeling), *F*(1,127) = 8.14, *p* < .001, η_*p*_^2^ = 0.06. Here, the lack of choice seemingly exacerbating Modeled-SSQ scores in the Inconsistent condition.

### Summary

As hypothesized, a main effect of the contrast assessing social modeling was significant. Contrary to hypotheses, however, the generalization effect (i.e., Consistent vs. Inconsistent Modeling) interacted with Choice. In the conditions without the intervention (i.e., the No Choice conditions), watching the confederate undertake a different VR activity exacerbated cybersickness relative to the Social Modeling Consistent condition where the confederate undertook the same VR activity (a significant difference between the No Choice/Consistent and No Choice/Inconsistent groups was confirmed in exploratory analysis; see Supplementary Materials 5). This contradicted expectations, as generalization to a novel stimulus (independent of choice) is typically similar, if not weaker. Therefore, this unanticipated finding warranted further investigation.

## Experiment 2

Given the unanticipated pattern of exploratory results concerning the generalization of social modeling found in Experiment 1, which was both complicated by the interaction with the factor of Choice and in an unexpected direction with respect to the No Choice condition specifically, a second study was conducted in an attempt to clarify these findings [47]. Experiment 2 is, therefore, a direct replication of the Social Modeling conditions excluding Choice to provide a clearer test of generalization without introducing the complicating factor of the interaction with the intervention. Choice was also omitted from this experiment to limit the burden on resources and maximize power to detect any general effect of social modeling independent of an additional manipulation.

### Methods

Data collection occurred between April 7 and July 13, 2022.

#### Participants

The sample (*N* = 78) comprised of 30 males, 46 females, and 2 of other gender identities ranging from 17 to 50 years of age (*M* = 31.09, *SD* = 7.96).

#### Design and procedure

A one way (Social Modeling: Consistent, Inconsistent, No Social Modeling) between-subjects design was employed. All participants underwent the No Choice procedure as described in Experiment 1.

#### Materials and measures

All methods and materials are consistent with Experiment 1.

#### Power and data analysis

A minimum of 26 participants per-group were required to achieve 80% power (α = 0.05) with an effect size of η_*p*_^2^ = 0.13 (based on the main effect of social modeling found in Experiment 1). Exclusions were extreme baseline cybersickness (*N* = 2), technical difficulties (*N* = 1), not following instructions (*N* = 6), withdrawal (*N* = 1), or failure of the manipulation check (*N* = 1).

### Results

Figure 1 depicts the group means of the primary outcome. The main ANCOVA model revealed that there was no main effect of the covariate, gender *F*(2,73) = 0.48, *p* = .62, η_*p*_^2^ = 0.01. There was no significant main effect of social modeling *F*(2,73) = 2.79, *p* = .07, η_*p*_^2^ = 0.07. However, planned orthogonal contrasts revealed that Modeled-SSQ scores were elevated in social modeling groups, compared with the No Social Modeling group *F*(1,73) = 5.00, *p* = .03, η_*p*_^2^ = 0.06. However, there was no significant difference in Modeled-SSQ scores between the Consistent and Inconsistent group, *F*(1,73) = 0.44, *p* = .51, η_*p*_^2^ = 0.006.

### Summary

In contrast to Experiment 1, those in the Inconsistent (No Choice) group did not experience exacerbated levels of cybersickness relative to the Consistent (No Choice) group (see Fig. 1). Instead, their SSQ scores were comparable to the Social Modeling Consistent group. Combined, both sets of results provide novel evidence of generalization of socially acquired nocebo effects, whereby the model and observer do not need to undertake identical interventions to induce a socially acquired nocebo effect relative to control. However, in Experiment 1, choice was found to reduce cybersickness in the Inconsistent group. It is unclear whether this difference was driven specifically by the exacerbated scores observed among those in the No Choice/Social Modeling Inconsistent condition in Experiment 1, or were associated with the Inconsistent environment more generally (i.e., would have still been observed in Experiment 2 had Choice been tested).

## Experiment 3

The discrepancy in results between Experiments 1 and 2 (i.e., whether the Inconsistent condition exacerbates symptoms) calls into question the effect of choice found in the Inconsistent group in Experiment 1. A final experiment was therefore conducted to assess the presence of a differential effect of choice between Social Modeling Consistent and Inconsistent groups, and to confirm that cybersickness elicited in the Inconsistent group is at least similar, if not greater, than the Social Modeling Consistent group. As the control (No Social Modeling) groups in both Experiments 1 and 2 revealed consistently low levels of cybersickness compared with the Social Modeling groups—establishing a robust effect of social modeling consistent with the literature [3, 4, 13, 35], Experiment 3 omitted this condition to minimize burden on resources.

### Methods

Small adjustments to refine the method and analyses were preregistered (AsPredicted #103304). Data collection occurred between July 26 and October 9, 2022.

#### Participants

The sample (*N* = 124) comprised of 60 males, 60 females, and 4 of other gender identities ranging from 18 to 54 years of age (*M* = 31.91, *SD* = 6.60).

#### Design and procedure

A 2(Social Modeling: Consistent, Inconsistent) × 2(Choice: No Choice, Choice) between-subjects design was employed. All participants underwent the Social Modeling procedure as described in Experiment 1.

#### Secondary outcomes: materials and measures

Given that no effect of choice on control was observed in Experiment 1 (see Supplementary Materials 4), the measure was adapted to a single item with greater face validity: “How in control do you feel right now?” rated on a VAS from 0 (Not at all), 50 (Moderately) to 100 (Very much). Extra memory questions were included to reinforce the cover story, and participants were asked to rate how positive an experience they perceived the social model to have had during the VR, and to what extent they perceived the social model to have experienced cybersickness.

#### Power and data analysis

A total of 31 participants per group (124 total) were recruited, powered to detect an effect of η_*p*_^2^ = 0.06, with α = 0.05 with 80% power. The effect size is derived from the interaction effect of choice and social modeling observed in Experiment 1. Exclusions were: extreme baseline cybersickness (preregistered as ≥40 sum score baseline SSQ or any single item ≥8, *N* = 9), technical difficulties (*N* = 6), not following instructions (*N* = 1), withdrawal (*N* = 1), or failure of the manipulation check (*N* = 1).

### Results

Figure 1 depicts the group means for the primary outcome. The ANCOVA model revealed that there was a main effect of the covariate gender, *F*(2,118) = 5.45, *p* = .005, η_*p*_^2^ = 0.08. However, there was no significant main effect of choice *F*(1,118) = 0.06, *p* = .81, η_*p*_^2^ < 0.001 or social modeling (Consistent vs. Inconsistent) *F*(1,118) = 0.01, *p* = .91, η_*p*_^2^ < 0.001 and no significant social modeling × choice interaction *F*(1,118) = 0.08, *p* = .78, η_*p*_^2^ < 0.001 on Modeled-SSQ scores.

Correlations were run between Modeled-SSQ scores and whether the confederate: (i) was perceived as experiencing cybersickness, and (ii) was perceived as having a positive experience. Neither correlation was statistically significant (both *p*s > .05).

### Summary

Contrary to the results of Experiment 1, no interaction between choice and social modeling was observed. This suggests that the difference reported in Experiment 1 was driven specifically by the exacerbated scores observed among those in the No Choice/Social Modeling Inconsistent condition, which was not replicated in Experiment 2, rather than via choice itself. Consistent with the results of Experiment 2 was the lack of difference in cybersickness elicited between the two Social Modeling groups. This suggests that at a minimum, the Inconsistent group experienced similar levels of cybersickness due to social modeling as the Consistent group, thereby providing consistent novel evidence across the three experiments that socially acquired nocebo effects do generalize.

## Pooled Analysis

Results from all experiments were combined for a pooled analysis. Table 1 summarizes the number of pooled participants in each experimental condition. To explore the main effects of Social Modeling and Choice, and their interaction, the analysis was conducted as a 2(Modeling Type: Consistent, Inconsistent) × 2(Choice: No Choice, Choice) + 1(No Social Modeling: both Choice and No Choice groups) ANCOVA controlling for gender as a covariate. There were no significant differences between experiments in the demographics (gender, age, and VR experience) of participants—see Supplementary Materials 5.

**Table 1:** Count of Participants in Each Condition Across Experiments

Social modeling condition	Choice condition	Experiment 1	Experiment 2	Experiment 3	Total
No Social Modeling	No Choice	20	26	0	46
	Choice	23	0	0	23
Social Modeling Consistent	No Choice	22	26	31	79
	Choice	23	0	31	54
Social Modeling Inconsistent	No Choice	23	26	31	80
	Choice	23	0	31	54
Total (*N*)		134	78	124	336

### Primary Outcome: Modeled-SSQ

Controlling for gender (*F*(2,332) = 5.21, *p* = .005, η_*p*_^2^ = 0.04), participants assigned to the experimental conditions (Modeling type: Consistent Choice, Consistent No Choice, Inconsistent Choice, Inconsistent No Choice) combined had significantly higher Modeled-SSQ scores (*M* = 4.50, *SE* = 0.74) relative to those in the control group (No Social Modeling Choice and No Social Modeling No Choice; *M* = 1.11, *SE* = 0.99) *F*(1,332) = 23.21, *p* < .001, η_*p*_^2^ = 0.07.

Figure 1 depicts the pooled group means. There was a nonsignificant main effect of Choice (Choice vs. No Choice), a nonsignificant effect of Modeling Type (Consistent vs. Inconsistent), and no interaction between Modeled Content and Choice. In summary, social modeling of any type (Consistent or Inconsistent) increased symptoms of cybersickness relative to No Social Modeling, while choice had no effect on socially acquired cybersickness.

### Secondary Outcomes: Expectancy, State Anxiety, VR Anxiety, Control, and Affect

**Fig. 2.**
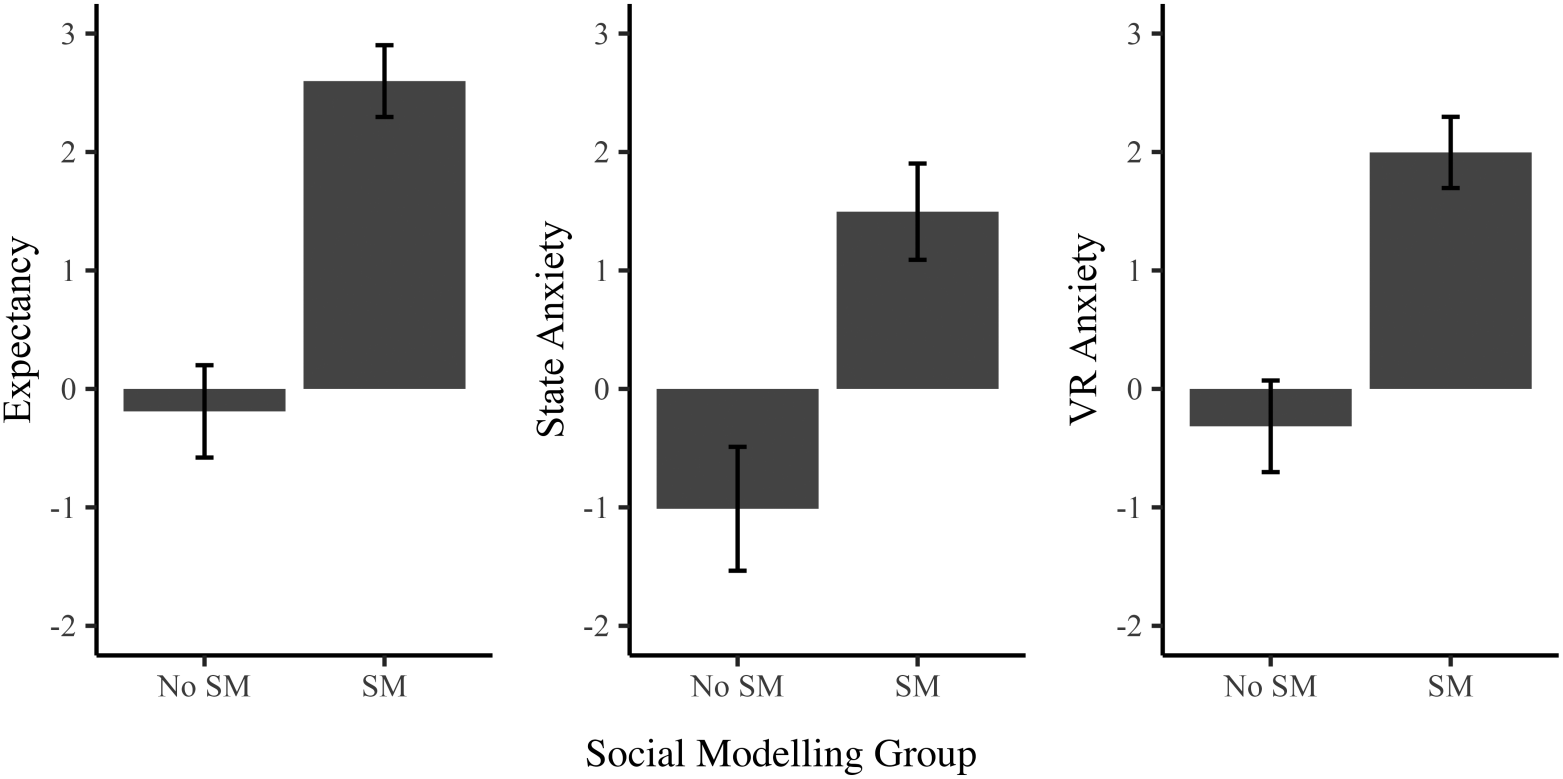
Mean baseline-adjusted expectancy, state anxiety, and VR anxiety: Social Modeling versus No Social Modeling. *Note.* All error bars are ± 1 SEM and account for the covariate (gender). *VR* Virtual Reality.

To investigate the potential underlying causes of the observed group differences in cybersickness between Social Modeling and No Social Modeling Groups, three one-way between-subjects ANCOVAs were conducted with expectancy, state anxiety, VR anxiety as the dependent variable, Social Modeling (No Social Modeling vs. Social Modeling: Consistent and Inconsistent combined) as the independent variable, and gender as the covariate. Please note that the same analyses for each separate study are presented in Supplementary Materials 5. Group means from the pooled analysis are depicted in Fig. 2. The pattern of results was similar across all three secondary outcomes (with full statistics reported in Table 2). There was a significant effect of the covariate gender on both state anxiety (*p* < .001) and VR anxiety (*p* = .001) but no significant effect of gender on expectancy (*p* = .17). Controlling for the covariate, Social Modeling significantly increased all three outcomes, relative to the No Social Modeling groups (all *p*s < .001). Each Social Modeling group (i.e., Consistent and Inconsistent) was also compared separately to the No Social Modeling group, and the pattern of results remains similar (see: https://osf.io/w2xcp/).

**Table 2:** ANCOVA Results (Contrast: No Social Modeling vs. Social Modeling Groups)

	*F*(1,332)	*p*	η_*p*_^2^
Expectancy	93.90	<.001	0.22
State anxiety	41.95	<.001	0.11
VR anxiety	65.20	<.001	0.16

*VR* Virtual Reality.

As predicted by the literature, Modeled-SSQ was significantly positively correlated with expectancy, *r*(334) = .23, *p* < .001, state anxiety, *r*(334) = .24, *p* < .001, and VR anxiety, *r*(334) = .28, *p* < .001.

Choice and No Choice groups were compared on perceived control and positive affect scores, but these dimensions did not significantly differ between groups (all *p*s > .05; see Supplementary Material 5).

### Mediation Analyses

**Fig. 3.**
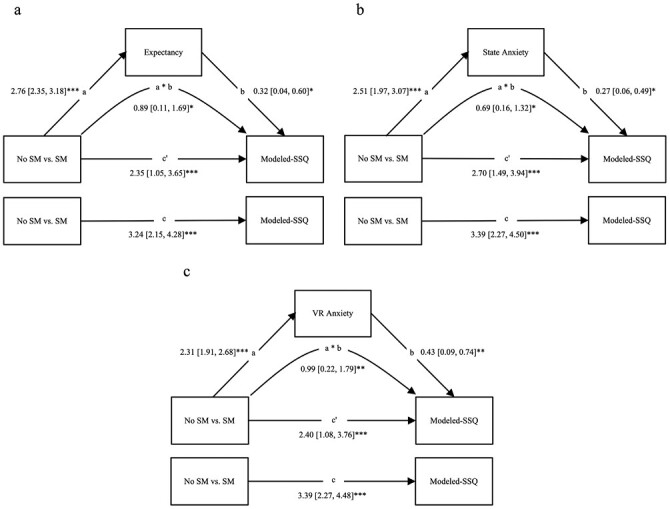
Mediatory effects of expectancy, state anxiety, and VR anxiety on cybersickness. *Note*. Values are unstandardized beta coefficients with 95% confidence intervals. Figures represent mediatory effects of expectancy (a), state anxiety (b), and VR anxiety (c). Covariate (gender) was included models b and c but is not represented here for brevity. Paths *a*, *b*, and *c* are the direct paths between variables. Curved path *a* × *b* is the indirect effect with bootstrapped CIs. Path *c*ʹ is the direct association between the group contrast and cybersickness, controlling for the indirect effect. *CIs* confidence intervals; *VR* Virtual Reality.

As presented in Fig. 3, three mediation analyses were conducted, with Social Modeling (No Social Modeling vs. Social Modeling: Consistent and Inconsistent combined, collapsed across choice) as the independent variable, Modeled-SSQ as the dependent variable, and expectancy, state anxiety, and VR anxiety, as separate mediators. Gender was included as a covariate in models where there was a significant effect (i.e., state anxiety and VR anxiety, see above). A very small number of participants in each cell self-reported their gender as “other” across the three experiments (*n* = 6; 1.79%), these participants were excluded from the mediation analyses that included gender as a covariate for the model to converge. Bootstrapping with 10,000 samples was conducted to determine 95% confidence intervals (CIs) which were used to determine significance.

#### Expectancy

Expectancy significantly mediated the effect of group on Modeled-SSQ score, direct effect = 2.35, 95% CI [1.05, 3.65], *p* < .01 and indirect effect = 0.89, 95% CI [0.11, 1.69], *p* = .02.

#### State anxiety

State anxiety significantly mediated the effect of group on Modeled-SSQ score, direct effect = 2.70, 95% CI [1.49, 3.94], *p* < .001 and indirect effect = 0.69, 95% CI [0.16, 1.32], *p* = .01.

#### VR anxiety

VR anxiety significantly mediated the effect of group on Modeled-SSQ score, direct effect = 2.40, 95% CI [1.08, 3.76], *p* < .01 and indirect effect = 0.99, 95% CI [0.22, 1.79], *p* < .01.

### Summary

The pooled results from the three experiments provide compelling evidence for an effect of social modeling in producing nocebo effects. Most importantly, they showed that social modeling can produce nocebo effects regardless of whether the experience witnessed is identical or similar to the one subsequently encountered. That is, that socially acquired nocebo effects can generalize from a specific observed experience to other experiences. Social modeling exacerbated expectancies regarding cybersickness and increased anxiety, and both expectancies and anxiety were found to mediate the relationship between social modeling and cybersickness. Choice, however, was not found to be an effective intervention to reduce socially elicited cybersickness.

## General Discussion

The present study explored whether socially modeled nocebo effects would generalize across different experiences and whether choice reduced these effects. There was consistent evidence for the effect of social modeling on cybersickness. Critically, this socially acquired nocebo effect occurred irrespective of whether the participant observed the model undergo an identical or distinct VR activity, indicating that socially acquired nocebo effects can generalize. There was, however, no consistent evidence that choice could inhibit these socially acquired nocebo effects.

Social modeling has been established as a powerful determinant of a range of symptoms [4, 7–10, 36, 37]. The present study revealed that social modeling plays an equally important role with respect to nocebo cybersickness [35]. As hypothesized, witnessing a social model experience cybersickness due to VR immersion exacerbated participants own experience of cybersickness compared with those that did not view the model. However, prior research has never investigated the effect of modeling *similar* interventions [6]. The present study therefore investigated the generalization of socially modeled symptoms beyond identical model/observer experiences. A key novel finding here was that the strength with which social modeling induced nocebo cybersickness did not depend on the similarity between the model and observers experience—that is, the social model did not have to undertake the exact same VR activity as the participant to elicit comparable levels of cybersickness. This novel finding has concerning implications, whereby social modeling may be significantly more widespread than previously imagined. Further research is needed to determine to what extent symptoms can spread, particularly in clinical settings.

Contrary to hypotheses, choice did not reduce symptoms. Given that choice was not found to enhance perceived control or engender positive affect, the manipulation (i.e., choice of sunny vs. snowy weather) may have lacked salience. It was important to hold the VR activity (i.e., the rollercoaster) constant to control for differences in cybersickness elicited. However, a more salient choice like choice of VR activity, or a greater number of choices overall, may have made for a more effective intervention. An important caveat to this limitation is to note that the choice provided to participants in the present study was not dissimilar to past research that yielded positive results in the context of explicit instruction and classical conditioning [26, 29]. As such, it is equally possible that our measures of control and affect themselves either lacked specificity or were not associated with the choice manipulation. For example, Tang et al. [48] found an effect of choice on conditioned placebo analgesia but failed to find a corresponding increase in perceived control, suggesting that these two constructs may be orthogonal. An alternative explanation is that choice may have differential effects based on the mode of induction, with socially modeled nocebo effects being especially resistant to attenuation. Another potential is for the effect of control to be dependent on culture, however the culture of the present sample is consistent with the cultures represented by past research [26]. The present study was the first to date to investigate choice with respect to social modeled nocebo effects, with past research exclusively concerning explicit instruction [16]. Clearly further investigation, employing experimental paradigms concerning choice that have previously been demonstrated to modulate the nocebo effect, are needed to elucidate the role of choice on socially acquired nocebo effects.

To date, empirical evidence addressing the mechanisms hypothesized to generate socially modeled nocebo effects has been both limited [1] and inconsistent [8, 10, 35, 37]. The present study addressed this concern in a large sample. Consistent with previous research [35, 37], expectancies and anxiety appear key to facilitating socially acquired nocebo effects. Both were found to be elevated after social modeling and to mediate the effect of social modeling on cybersickness. While no effect of choice was observed in the present study, the strength of identifying these mediators is that they can be employed to inform future targeted interventions. As such, research may wish to focus on these mechanisms when developing methods to attenuate socially acquired nocebo effects.

The present study demonstrated several novel results based on a large representative sample. However, limitations must be noted. First, the study was conducted single-blind, which may have led to experimenter bias. However, care was taken to ensure that all instructions were delivered by the experimenter using identical scripts across experimental sessions. Furthermore, the social model was a pre-recorded video to ensure consistency, and all questionnaires completed by participants were administered remotely via Qualtrics to reduce biased responding. Second, the study was conducted entirely online via Zoom meaning the quantity of social information conveyed was dependent on the strength of participant’s internet connection and limited to what was observable via webcam, introducing experimental noise. Testimony to the strength of social modeling, strong effects were found irrespective of whether the VR environment was consistent or inconsistent, despite these technical limitations. However, social modeling in live settings may be stronger and potentially more receptive to manipulations such as choice. Third, the social model was a male and as such the effect of the model’s gender remains unexplored. Furthermore, any gender effects reported may be confounded with gender match between participant and model. Finally, the manipulation check was general. As such, it is possible that the number of participants that recognized the specific purpose of the studies may be underrepresented.

Evidence of generalization found in the present study is highly problematic with respect to both clinical and nonclinical settings. Results extend previous research, demonstrating empirically for the first time that socially modeled nocebo effects are not limited to identical interventions. People do not have to observe others experience an identical intervention for social transmission to occur, meaning symptoms could potentially spread between different brands of similar medication, between similar medical procedures or between similar experiences like VR which is increasingly being used in clinical contexts. This suggests that opportunity for social modeling may be significantly more widespread than previously imagined. This emphasizes the importance of future research into interventions to reduce these negative health effects. Beyond choice, potential avenues that remain unstudied with respect to socially modeled symptoms include: side-effect framing [17, 18], nocebo education [19, 20], latent inhibition [23], and affect manipulations [25]. Importantly, the present study identified negative expectancies and anxieties key mechanisms, meaning that interventions targeting these mechanisms are likely to be most effective. Further exploration of the generalization of social information is also warranted with respect to different symptom outcomes (e.g., pain, headache, insomnia) within different contexts (e.g., clinically). In addition, future research should investigate to what extent these socially modeled effects can generalize, for example between more dissimilar stimuli (VR rollercoaster vs. VR walk on the beach), across modes of nocebo induction (nausea induced via VR vs. GVS) or, if placebo effects elicited via social modeling can generalize in a similar way.

In summary, results of this study demonstrate that social modeling is a powerful determinant of nocebo effects, with the potential to impact our experiences in both a robust and diffuse manner. As choice was not found to reduce these effects at all, further research concerning negative expectancies and anxieties is critical to reduce the harm that can arise from these socially acquired side-effects.

## Supplementary Material

kaad056_suppl_Supplementary_Materials

## Open Science Transparency Statements

1. The study was preregistered at AsPredicted.org: https://aspredicted.org/FZN_AFT (Experiments 1 and 2) https://aspredicted.org/HXH_H48 (Experiment 3)

2. The analysis plan was registered prior to beginning data collection at AsPredicted.org: https://aspredicted.org/FZN_AFT (Experiments 1 and 2) https://aspredicted.org/HXH_H48 (Experiment 3)

3. Deidentified data from this study are available in a public archive: https://osf.io/w2xcp/

4. Analytic code used to conduct the analyses presented in this study are available in a public archive: https://osf.io/w2xcp/

5. Some of the materials used to conduct the study are presented in a public archive: https://osf.io/w2xcp/
