# Supplementary material for: Socially Acquired Nocebo Effects Generalize but Are Not Attenuated by Choice
Source: Ann Behav Med. 2023 Sep 27;57(12):1069–80. doi: 10.1093/abm/kaad056 (PMC10653279; doi:10.1093/abm/kaad056)
Supplement: kaad056_suppl_Supplementary_Materials [file kaad056_suppl_supplementary_materials.docx]

**Electronic Supplementary Material 1**

##### *CONSORT Flow Diagram of the Participant Recruitment Process Experiment 1*

##### *CONSORT Flow Diagram of the Participant Recruitment Process Experiment 2*

##### *CONSORT Flow Diagram of the Participant Recruitment Process Experiment 3*

**Electronic Supplementary Material 2**

*Description of yoking procedure*

Participants were first stratified by self-report gender. The first participant (of a specified gender) was assigned to the Choice Condition and randomised to one of the three Social Modelling conditions. The second was yoked to the ‘No Choice’ condition of the same Social Modelling counterpart. The third was assigned to the Choice condition and randomised to one of the two remaining Social Modelling Conditions. The fourth was yoked to the No Choice Condition of the same Social Modelling counterpart. Finally, the fifth participant was assigned to the Choice Condition of the remaining Social Modelling Condition and the sixth participant yoked to the final No Choice Condition. This was performed for each sextuplet in order of their participation.

**Electronic Supplementary Material 3**

*Screenshot of the Sunny (top) and Snowy (bottom) rollercoaster experienced by all participants*

*
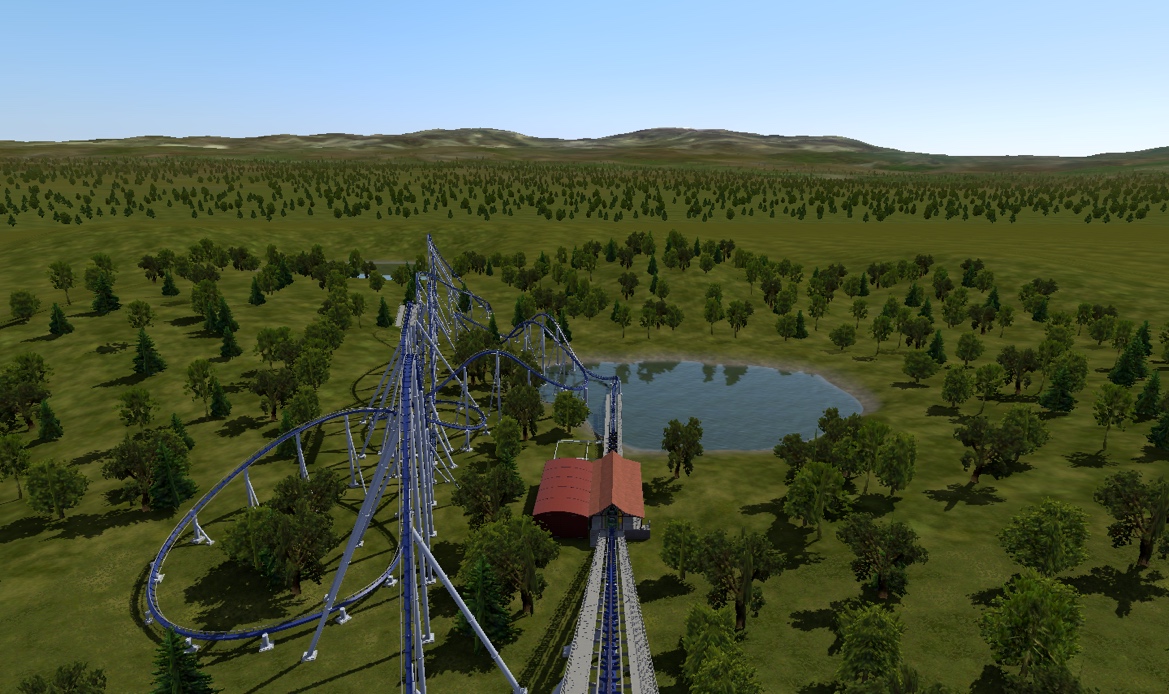

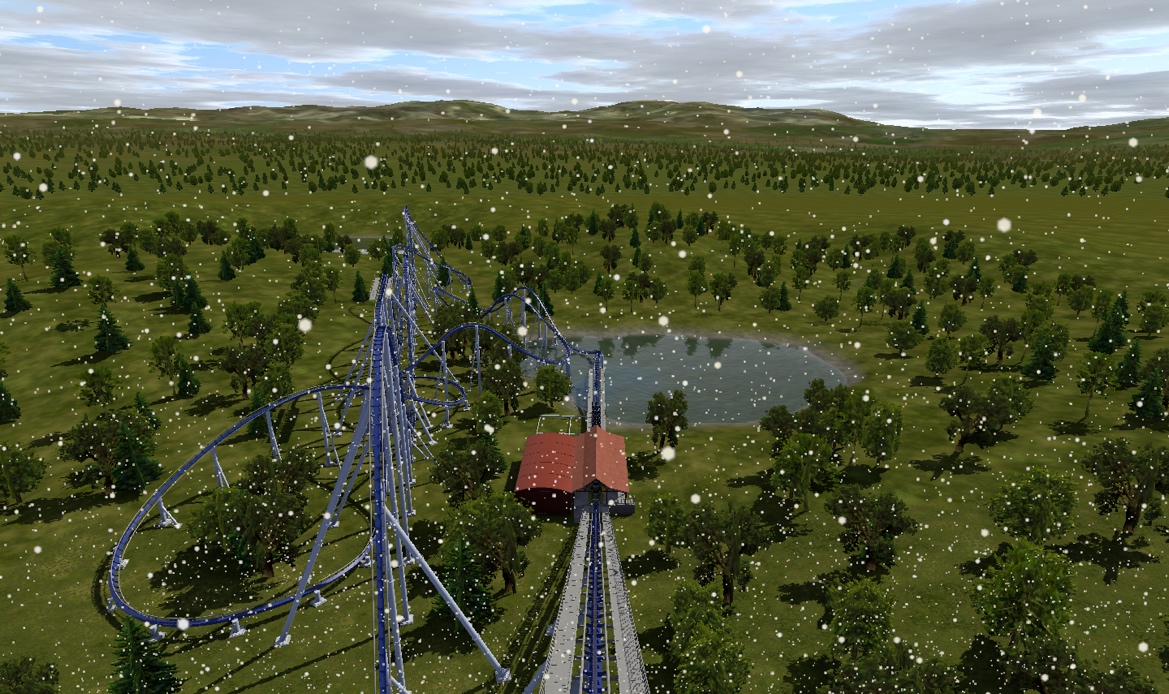
*

**Electronic Supplementary Material 4**

*Flow Chart of the Experimental Procedure*


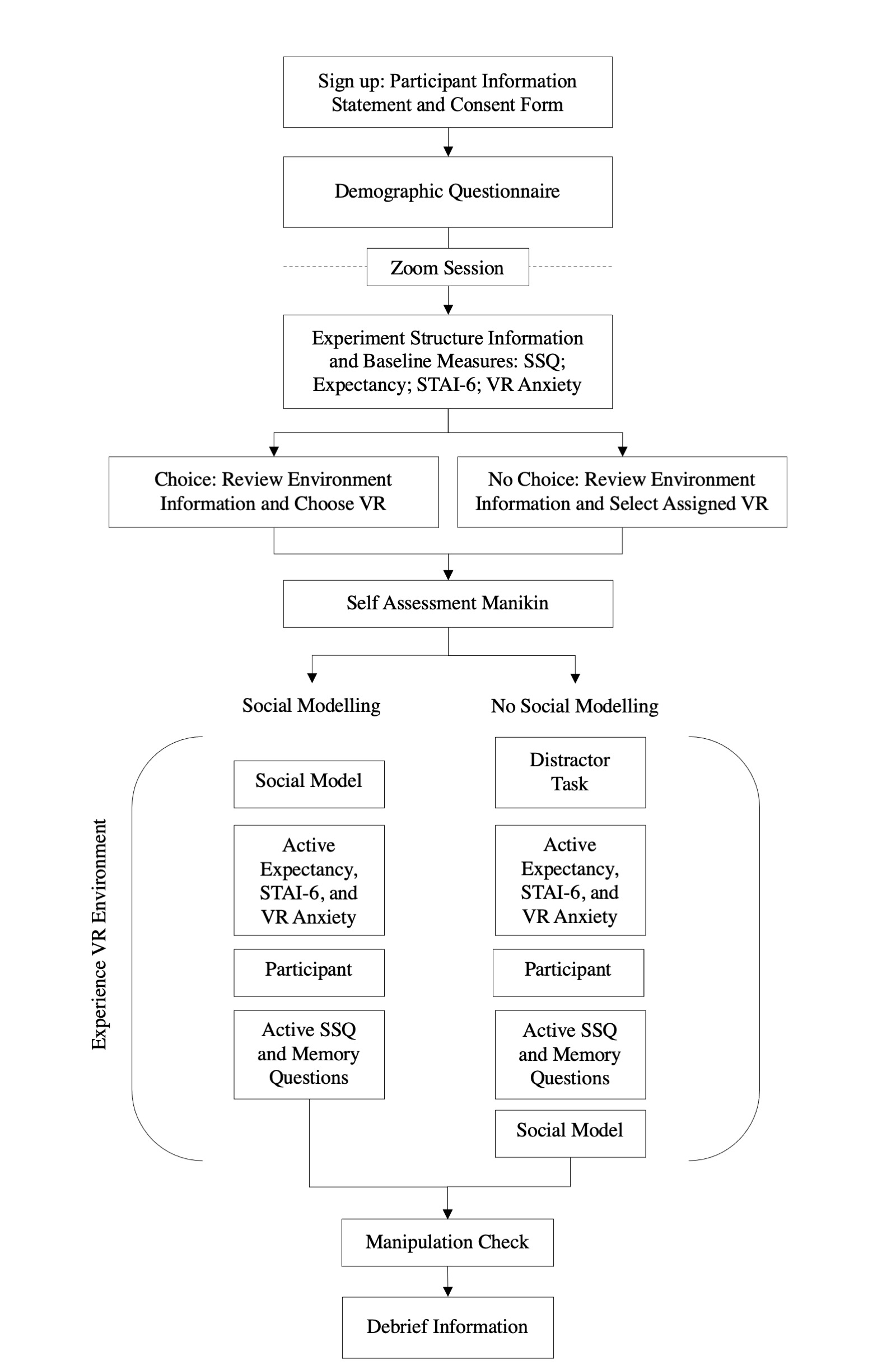


**Electronic Supplementary Material 5**

Supplementary Statistical Analyses

Full statistical analyses, group means, graphical summaries and accompanying code are available at: <https://osf.io/w2xcp/?view_only=cafd0e83882844699fd572e6b08d1302>.

**Table 1**

*Reliability Statistics (Cronbachs’ $\alpha$)*

|  | Modelled SSQ | Full SSQ | STAI-6 |
| --- | --- | --- | --- |
| Experiment 1 | .83 | .89 | .71 |
| Experiment 2 | .82 | .89 | .65 |
| Experiment 3 | .81 | .90 | .77 |

**Table 2**

*Analysis of Baseline Characteristics (Quantitative) Across Experiments*

|  | Experiment 1 | | Experiment 2 | | Experiment 3 | |
| --- | --- | --- | --- | --- | --- | --- |
|  | *F*(5,128) | *p* | *F*(2,75) | *p* | *F*(3,120) | *p* |
| Age | 1.05 | .39 | 0.33 | .72 | 0.62 | .61 |
| Modelled-SSQ | 1.30 | .27 | 0.28 | .76 | 0.59 | .62 |
| Full-SSQ | 0.44 | .82 | 0.57 | .57 | 0.66 | .58 |
| Expectancy | 0.74 | .60 | 2.53 | .09 | 1.06 | .37 |
| STAI-6 | 0.53 | .75 | 0.99 | .37 | 0.97 | .41 |
| VR Anxiety | 0.95 | .45 | 0.40 | .67 | 0.17 | .92 |

*Note.* Given all baseline characteristics were measured prior to any choice or social modelling manipulation, One-Way ANOVAs were conducted to assess differences in baseline characteristics between the groups.

**Table 3**

*Analysis of Baseline Characteristics (Qualitative) Across Experiments*

|  | Experiment 1 | | Experiment 2 | | Experiment 3 | |
| --- | --- | --- | --- | --- | --- | --- |
|  | $\chi^{2}$(5, *N* = 134) | *p* | $\chi^{2}$(2, *N* = 78) | *p* | $\chi^{2}$(3, *N* = 124) | *p* |
| Gender | 0.14 | 1.00 | 7.27 | .12 | 2.13 | .91 |
| VR Experience | 4.62 | .46 | 1.97 | .37 | 1.90 | .59 |

*Post-hoc: Social Modelling Consistent vs Inconsistent (No Choice groups only).* A two-sided independent samples t-test revealed that the Inconsistent Social Modelling group had significantly higher Modelled-SSQ scores (*M*=8.52, *SD*=5.81) than the Consistent group (*M*=3.68, *SD*=4.81), *t*(43)=-3.03, *p*=.004, *d*=0.90.

*Effect of Control*. In Experiment 1, there was no significant difference in perceived control as measured by the SAM between Choice (*M*=0.15, *SD*=0.81) and No Choice groups (*M*=0.00, *SD*=1.09), *t*(132)=-0.91, *p*=.18, *d*=0.16.

*Between experiment demographics differences.* There was no significant difference between experiments with respect to gender, $\chi^{2}$(4, *N* = 336)=6.97, *p*=.14, VR experience, $\chi^{2}$(2, *N* = 336)=1.70, *p*=.43, or age of participants, *F*(2,333)=0.61, *p*=.54.

Secondary Outcomes: Expectancy, State Anxiety, VR Anxiety, Control and Affect

**Table 4**

*Experiment 1 ANCOVA results (No Social Modelling vs Social Modelling Groups)*

|  | No Social Modelling  *M* | Social Modelling  *M* | *F*(1,127) | *p* | $\eta_{p}^{2}$ |
| --- | --- | --- | --- | --- | --- |
| Expectancy | -0.02 | 2.22 | 36.24 | <.001 | 0.22 |
| State Anxiety | 0.42 | 2.75 | 20.37 | <.001 | 0.14 |
| VR Anxiety | 0.25 | 2.31 | 29.46 | <.001 | 0.19 |

**Table 5**

*Experiment 2 ANCOVA results (No Social Modelling vs Social Modelling Groups)*

|  | No Social Modelling  *M* | Social Modelling  *M* | *F*(1,73) | *p* | $\eta_{p}^{2}$ |
| --- | --- | --- | --- | --- | --- |
| Expectancy | -0.18 | 2.70 | 29.86 | <.001 | 0.29 |
| State Anxiety | -1.27 | 1.26 | 17.08 | <.001 | 0.19 |
| VR Anxiety | -0.10 | 2.42 | 27.30 | <.001 | 0.27 |

**Table 6**

*Baseline-adjusted Expectancy, Anxiety and VR Specific Anxiety for each group*

| Social Modelling Condition | Choice Condition | Expectancy | | STAI | | VR Anxiety | |
| --- | --- | --- | --- | --- | --- | --- | --- |
|  |  | *M* | *SE* | *M* | *SE* | *M* | *SE* |
| No Social Modelling | No Choice | -0.43 | 0.43 | -1.18 | 0.58 | -0.43 | 0.43 |
|  | Choice | 0.26 | 0.53 | -0.66 | 0.72 | -0.03 | 0.53 |
| Social Modelling Consistent | No Choice | 2.64 | 0.37 | 1.53 | 0.5 | 2.12 | 0.37 |
|  | Choice | 2.31 | 0.40 | 1.65 | 0.54 | 1.84 | 0.40 |
| Social Modelling Inconsistent | No Choice | 2.68 | 0.35 | 1.44 | 0.47 | 1.95 | 0.34 |
|  | Choice | 2.69 | 0.41 | 1.39 | 0.56 | 2.13 | 0.41 |

*Note.* Results are averages across the covariate gender. By dividing the results of the analysis by group the pattern of results does not differ, all social modelling groups experience higher levels of expectancy, state anxiety and VR specific anxiety.

**Table 7**

*Expectancy, State Anxiety and VR Anxiety Aggregated by Gender*

| Gender |  | Expectancy | | STAI-6 | | VR Anxiety | |
| --- | --- | --- | --- | --- | --- | --- | --- |
|  | *N* | *M* | *SE* | *M* | *SE* | *M* | *SE* |
| Female | 172 | 2.05 | 0.17 | 2.36 | 0.23 | 2.12 | 0.17 |
| Male | 158 | 1.61 | 0.18 | 1.36 | 0.24 | 1.36 | 0.18 |
| Other | 6 | 1.42 | 0.89 | -1.63 | 1.19 | 0.30 | 0.88 |

*Note.* Results are averaged across experimental groups.

*Effect of Choice.* See Table 8 for group means. There was no significant difference between Choice and No Choice groups in perceived control, *t*(334)=-1.47, *p* = .07, happiness, *t*(210)=0.01, *p* = .50, or arousal, *t*(210)=0.21, *p* = .41.

**Table 8**

*Group Means for Choice and No Choice Groups*

|  | No Choice | | Choice | |
| --- | --- | --- | --- | --- |
|  | *M* | *SD* | *M* | *SD* |
| Perceived Control | -0.06 | 1.05 | 0.10 | 0.91 |
| Happiness | 6.94 | 1.37 | 6.94 | 1.37 |
| Arousal | 4.27 | 2.12 | 4.20 | 1.65 |

*Note.* Averaged across type of Social Modelling
